# A graph neural network framework for mapping histological topology in oral mucosal tissue

**DOI:** 10.1186/s12859-022-05063-5

**Published:** 2022-11-25

**Authors:** Aravind Nair, Helena Arvidsson, Jorge E. Gatica V., Nikolce Tudzarovski, Karl Meinke, Rachael. V Sugars

**Affiliations:** 1 Division of Theoretical Computer Science, Department of Computer Science, KTH Royal Institute of Technology, Stockholm, Sweden; 2 Division of Oral Diagnostics and Rehabilitation, Department of Dental Medicine, Karolinska Institutet, Stockholm, Sweden

**Keywords:** Digital pathology, Graph neural network, Tissue topology, Cell-graph, Convolutional neural network, Machine learning, Oral mucosa

## Abstract

**Background:**

Histological feature representation is advantageous for computer aided diagnosis (CAD) and disease classification when using predictive techniques based on machine learning. Explicit feature representations in computer tissue models can assist explainability of machine learning predictions. Different approaches to feature representation within digital tissue images have been proposed. Cell-graphs have been demonstrated to provide precise and general constructs that can model both low- and high-level features. The basement membrane is high-level tissue architecture, and interactions across the basement membrane are involved in multiple disease processes. Thus, the basement membrane is an important histological feature to study from a cell-graph and machine learning perspective.

**Results:**

We present a two stage machine learning pipeline for generating a cell-graph from a digital H &E stained tissue image. Using a combination of convolutional neural networks for visual analysis and graph neural networks exploiting node and edge labels for topological analysis, the pipeline is shown to predict both low- and high-level histological features in oral mucosal tissue with good accuracy.

**Conclusions:**

Convolutional and graph neural networks are complementary technologies for learning, representing and predicting local and global histological features employing node and edge labels. Their combination is potentially widely applicable in histopathology image analysis and can enhance explainability in CAD tools for disease prediction.

## Background

Machine learning (ML) algorithms based on convolutional neural networks (CNNs) have made significant contributions to image analysis in digital pathology (DP). CNN algorithms have been shown to provide accurate computational solutions to low-level problems such as cell nucleus detection, as well as high-level problems such as gland segmentation and cancer classification, grading and survival prediction [1].

However, concept representations for histological features visible in the underlying digital tissue image, are missing in CNN models. Such features are often the basis for clinical grading schemes in pathology. The absence of histological features in CNN models renders their explanatory power weak, in terms a human pathologist could understand. Consequently, several researchers have considered graph neural networks (GNNs) as an alternative ML paradigm that can better fit the explanatory, clinical and regulatory needs of DP [2]. GNNs are capable of learning the properties and characteristics of a wide range of graph theoretic structures [3]. Labelled graph structures are widely used in mathematical modeling, as they are well suited to represent high-level conceptual features (e.g. semantic networks [4]). In fact, digital images are just a special case consisting of gridded 2-dimensional labelled graphs. Many efficient algorithms exist to construct and analyse graph models [5, 6].

An important class of graph models for tissue representation in the DP literature are cell-graphs, which have a lengthy history that predates modern deep learning technology [7]. The related literature summary of Additional file 1: Table S1 indicates research directions for this approach. Cell-graphs are motivated by the fact that cells in a tissue are not randomly organised, their spatial relationships are structured to allow the functions of the cells and the tissue. Changes in these relationships indicate a change in function, which reflects on health and disease. In a cell-graph we can explicitly represent the high- and low-level histological features as well as relationships seen in tissue, in terms of entities (termed nodes) and relations (termed edges). A typical node is the centroid of a cell nucleus which has visual attributes, including location, colour and texture, as well as histological attributes such as cell type and morphology. A typical relation between entities is distance. By modeling tissue in this precise but abstract way, a cell-graph has the potential to capture the distinctive topological^1^ properties characterising a wide range of histological features, even when these are spread over large regions of a tissue image. Several approaches to cell-graph modeling have been proposed for different tissue types, and the cell-graph model must be adapted to the task, tissue and disease (c.f. Groups I, II, IV of Additional file 1: Table S1). While histological features lack a precise (i.e. mathematical) description, they can in principle be machine learned as a graph concept from a sufficiently large set of positive and negative examples [8–10].

To support explainability and generate trust in ML predictions, it is important that cell-graphs are amenable to human analysis and retrospective confirmation of ML predictions against ground truth image data in cases of doubt. Many GNN solutions published in the literature have addressed DP problems, by providing an *end-to-end* (i.e. black-box) solution [2] (c.f. Groups III, IV and V of Additional file 1: Table S1). Such GNN solutions compute, for example a binary classification (diseased/non-diseased) of an entire cell-graph according to some clinical grading scale. This is done without any explicit identification or analysis of the diagnostic contribution of individual histological features. In such end-to-end approaches, explainability is weakened by missing concepts, which must be recovered by additional post-hoc explainability techniques, such as sub-graph masking [11, 12].

In our work, we consider an alternative to this end-to-end GNN approach, where GNNs locate, classify and quantify relevant histological features in cell-graph structures, that a clinical pathologist might have used, when guided by a specific diagnostic model. In ML terms, this represents the use of hand-crafted features for learning which come directly from the science of histology itself, rather than from visual attributes of a digital image. A key general question here is: *how well can we represent and learn high-level histological features using graph theory and GNNs*?

This report describes an ML pipeline for cell-graph representation and learning of histological features based on both node and edge feature analysis. We illustrate its application to the representation and ML of the basement membrane (BM) as part of a clinical grading model for oral mucosal lesions in patients post-haematopoietic stem cell transplantation in the form of chronic graft-versus host disease (cGvHD) [13]. The grading model has identified changes in the BM as a key feature in the histopathological disease process of oral mucosal cGvHD [13]. The BM is a high-level histological feature separating the epithelium from the underlying lamina propria. It plays a key role in mediating interactions between cells in many processes like development, healing, fibrosis and cancer [14]. We propose a model for representing this high-level feature by classifying edges in a cell-graph to identify the cellular interface between the two tissue types separated by the BM.

### Contributions and significance

We present a hierarchical approach to cell-graph construction based on a two stage pipeline that combines CNN-based analysis of local histological features with GNN-based analysis of global histological features. This algorithmic approach can be used to directly translate the organisation and features of a clinically-based disease model into a set of ML-based classification and localisation algorithms that include explicit concept representations. In this way, the rationale for ML predictions can be traced backwards starting from the clinical disease model through a set of concept representations and measurements to the original ground truth image data, in a way that a human pathologist could understand and confirm.

The main contributions of this work are:

1. An explanatory paradigm for ML-generated edge label predictions in cell-graphs that is anchored in well-defined histological phenomena, and that offers enhanced opportunities to compare ML predictions with ground truth data using pathologist expertise.
2. Novel concepts and results regarding the represention and ML of histological features using both node and edge label representations.
3. Applications of cell-graphs and GNNs in DP outside the widely studied domain of oncology.

## Methods

### Cell-graph model

A cell-graph *G* can be defined as a labelled undirected graph

$$\begin{aligned} G = (V, E, \lambda : V \rightarrow L_V, \varepsilon : E \rightarrow L_E), \end{aligned}$$

where *V* is a finite set of node (entity) names $v \in V$, and *E* is a finite set of edges (relations) $e \in E$. An undirected edge *e* can be represented as a pair (doubleton set) of nodes $e = \{ v_1, v_2 \}$.^2^ The function $\lambda : V \rightarrow L_V$ is a node labelling which maps each node $v \in V$ to a node label $\lambda (v) = l \in L_V$, and the function $\varepsilon : E \rightarrow L_E$ is an edge labelling which maps each edge $e \in E$ to an edge label $\varepsilon (e) = l' \in L_E$. In our approach, every cell-graph *G* is associated with an underlying digital image *I*, and *G* attempts to capture salient histological facts about *I* while abstracting away many irrelevant visual details.

In our approach to cell-graph modeling of the BM outlined below, a node label is a triple $l = (x, y, type)$ where $x, y \in {\mathbb {R}}$ represents the geometric co-ordinates of the centroid of a cell nucleus, while *type* takes one of the four discrete cell class names. Note that the geometric coordinates *x*, *y* are only used for visualising a cell-graph and superimposing it on a tissue image. They are not used for GNN training as they are orientation dependent. An edge label $l' \in L_E$ will be Boolean value (true/false) to indicate whether the edge crosses the BM or not. An edge $\{v_1, v_2\}$ implicitly indicates a proximity relation between nodes $v_1$ and $v_2$ in the underlying digital image *I*. In general, we can extend the label sets $L_V , L_E$ with any number of additional parameters to represent further histological features and structures in hierarchical grading schemes e.g. oral mucosal cGvHD [13]. However, the above parameters suffice to define a cell-graph model of the BM.

### Datasets

**Fig. 1:**
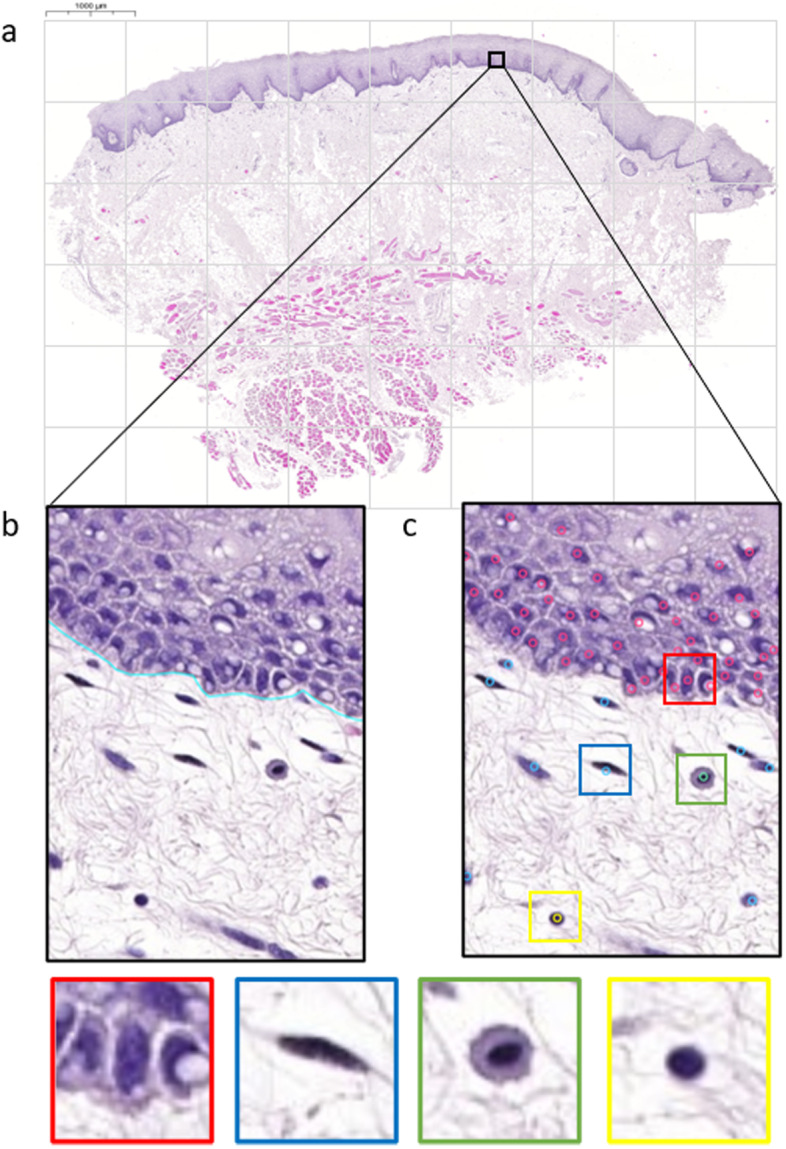
WSI of healthy buccal oral mucosa that has been segmented into tiles of $2000 \times 2000$ pixels (**a**). Annotation of the extension of the BM visualised with blue line (**b**). Nuclei centroids were annotated (**c**) and labelled as either epithelial (red), fibroblast or endothelial (blue), inflammatory (green) or lymphocytic (yellow)

Datasets for training CNN models of cell classification and localisation were created using whole slide images (WSI) of oral mucosal biopsies from nine patients receiving haematopoetic cell transplantation and four healthy controls [13]. Biopsies were obtained from patients attending the Department of Maxillofacial Surgery, Karolinska University Hospital or were retrieved retrospectively from Stockholm’s Medicine Biobank (Sweden Biobank). All patients were treated in accordance with the Helsinki Declaration. Previously, the biopsies had been sectioned and stained with haematoxylin and eosin (H &E) (Histolab Products AB, Gothenburg, Sweden), and scanned (x40) using a 3D Histech Midi Scanner System (3D Histech, Histolab Products AB). Sections were chosen to provide a wide variance of stain intensity and BM alterations. The 13 WSIs were visualised using CaseViewer (3D Histech, Histolab Products AB), annotated and exported (1$\mu m$ per 2 pixels), prior to segmentation into 152 tiles ($2000 \times 2000$ pixels) in .tiff format. 51 tiles were selected located close to the BM, of which 42 included both epithelium and lamina propria. All tiles were manually annotated by three histology experts to establish ground truth data regarding the location (i.e. centroid coordinates) and classification of cell nuclei. Cell classifications were identified as epithelial, fibroblast and endothelial, inflammatory or lymphocytic (Fig. 1). Both locations and cell classifications were annotated as meta-information for the .tiff tile files using JSON formatted data annotations created with an in-house graphical annotation tool. Table 1 summarises the distribution of cell classes in the dataset, and indicates data skew among these classes, especially in the inflammatory class, which is due to differing degrees of inflammation from healthy to severe oral cGvHD [13].

For GNN learning of the BM as a cell-graph concept, it was necessary to construct a set of ground truth cell-graph models showing the location and topological context of the BM. 42 tiles with BM were annotated by histology experts using spline segments to represent BM location. Below, we describe the automated process of translating the spline segment annotations into ground truth cell-graph edge labels. Table 2 shows the resulting distribution of edge classes in the ground truth cell-graph dataset. It indicates data skew between the two edge classes, as the BM involves only a small percentage of each tile area.

Both cell annotations and BM annotations were reviewed by two histology experts to implement a consensus-based quality control process. Thus, two sets of high-quality ground truth data were available for ML: cell type annotations and BM annotations.

**Table 1:** Distribution of cell classes in the dataset for training the CNN models for nuclei detection and classification

	Tiles	Epithelial	Fibroblasts and endothelial	Inflammatory	Lymphocytic	Total
CNN dataset—annotated nuclei						
Total	51	27,226	22,017	1809	11,676	62,728

**Table 2:** Distribution of edge classes in the dataset for training the GNN models for identifying the edges crossing the BM

	Tiles	Non-crossing	Crossing	Total
GNN dataset—annotated edges				
Total	42	161,991	10,123	172,114

### Experimental procedures

**Fig. 2:**
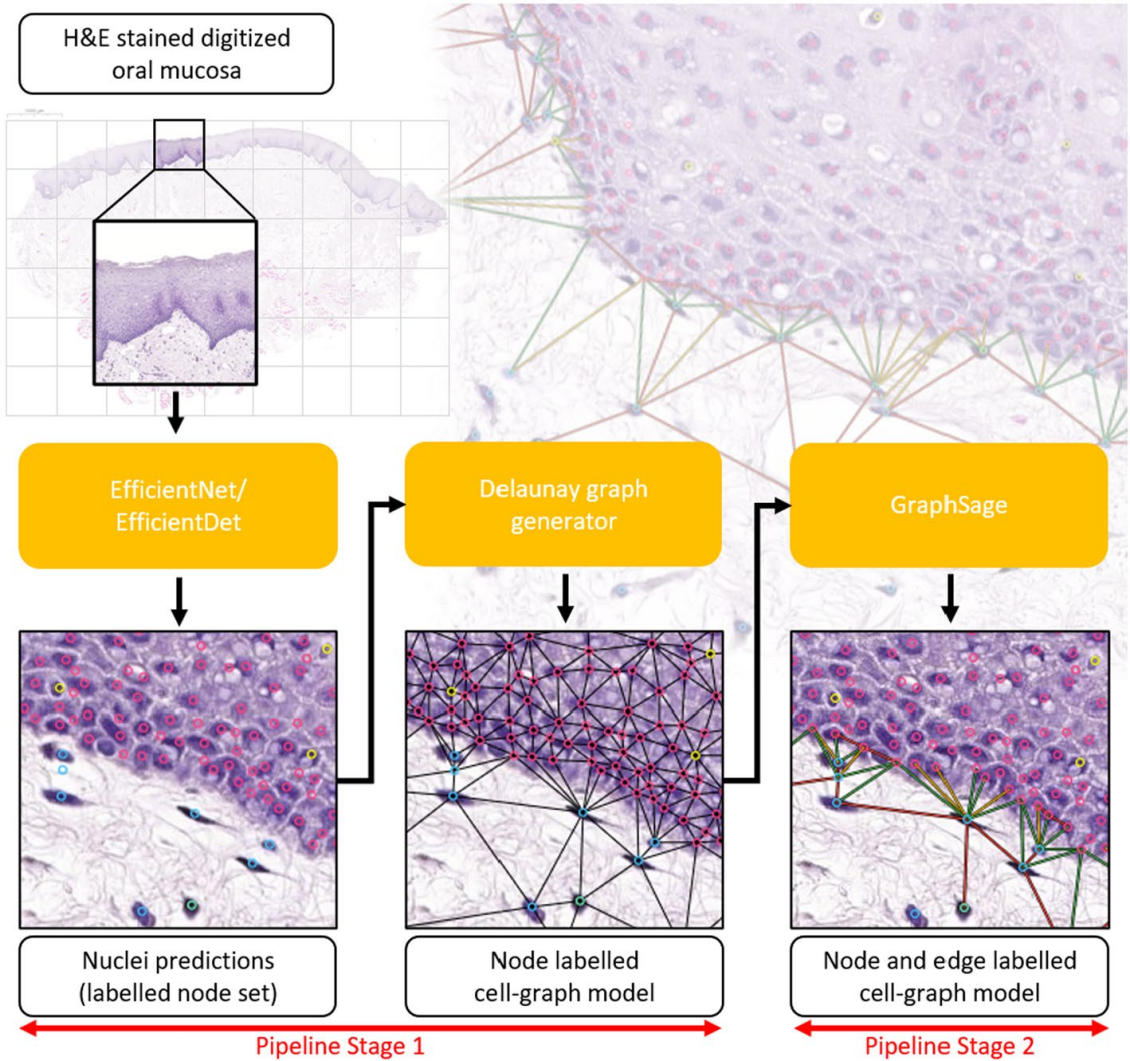
ML pipeline for cell-graph construction and histological feature prediction. Node labels are generated by our CNN model (as in Fig. 1) and a node-labelled cell-graph is constructed with Delaunay triangulation. Edges are labelled by our GNN model to produce a fully node and edge labelled cell-graph with both high- and low-level histopathological features. Predicted edge labels (crossing/not crossing the BM) are visualised as true positive (green), false negative (yellow) and false positive (red). True negative edges are not visualised

Figure 2 illustrates our ML-based architecture for the construction and analysis of cell-graphs starting from high-resolution digital images of H &E stained tissue. The figure shows the major software components and data flows from left to right in a two stage pipeline.

In Stage 1 of the pipeline, the input is a digitised image *I* of H &E stained tissue. This tissue image is analysed by a deep CNN to identify small localised histological features. We have focused on the histological classification of four common cell populations present in our tissue samples: inflammatory, lymphocyte, fibroblast/endothelial and epithelial. However, other cell classes could also be considered such as apoptotic or mitotic cells. For CNN-based image analysis, we used the EfficientNet algorithm [15] to predict all cell classifications, and the EfficientDet algorithm [16] to predict the location of the centroids of all cell nuclei. These state-of-the-art deep-learning CNN algorithms make efficient use of a limited number of training parameters, and the smallest architecture EfficientNet-B0 (5.3 million trainable parameters) already gave high quality cell recognition. Note that no hand-crafted features (such as textures, cell size or shape) or cell segmentation are used in Stage 1. Therefore, in principle, any other off-the-shelf deep CNN could be used in Stage 1, which makes this modular approach well adapted to future improvements in CNN algorithms. In particular, given larger ground truth annotated training sets and/or synthetic data, deeper versions of EfficientNet and EfficientDet could be used to give better CNN performance (e.g. EfficientNet-B1).

The CNN output of Stage 1 is a list of nodes $v_1, v_2 , \ldots , v_n$ and for each node $v_i$ a label prediction $\lambda (v_i) = (x_i, y_i, type_i)$. The node set $V = \{ v_1, v_2 , \ldots , v_n \}$ is thus the basis for a cell-graph model *G* of *I*.

The next step in Stage 1 is the addition of a suitable edge set *E* to the labelled node set *V* which was the output of the CNN. For this we use a *d*-distance^3^ limited Delaunay triangulation method [17]. Given the node set $V = \{ v_1, v_2 , \ldots , v_n \}$, Delaunay triangulation aims to connect three nodes $v_i, v_j, v_k \in V$ in a triangle, whenever the triangle’s circumcircle is void of any other node point in *V*. Commonly, the Delaunay triangulation of a node set is unique up to graph isomorphism. This situation ensures the consistency of cell-graph construction across different data sets. For triangulation no edges need cross one another, and the cell-graph is said to be *planar*. For generating the triangulation edge set *E*, we used the divide and conquer algorithm [18] that generates the edge set in ${\mathcal {O}}(n\log {}n)$ time. For *d*-distance Delaunay, we delete all Delaunay triangulation edges where the end nodes are separated by a distance greater than *d* in the image.

Thus, the final output of Stage 1 is a partially labelled cell-graph $G = (V, E)$ in which the node set *V* is defined and labelled (by $\lambda$), and the edge set *E* is defined but not yet labelled.

In Stage 2 we use a GNN algorithm to predict the edge label $\varepsilon (e) = l' \in L_E$ of each edge $e \in E$ as a Boolean value (true/false). The edge label $\varepsilon (e)$ of an edge $e = \{ v_1, v_2\}$ should be *true* if a straight line between the image coordinates $x_1, y_1$ of node $v_1$ and $x_2, y_2$ of node $v_2$ crosses the BM in the underlying tissue image *I*, otherwise $\varepsilon (e)$ should be *false*.

There are many different architectures for GNNs published in the ML literature (see e.g. the surveys [19–21]). Message passing GNNs are robust to changes in the graph size, and this robustness is essential for working with different tissue datasets. For the Stage 2 GNN algorithm we used GraphSage [22], which applies a basic form of message passing. In principle, many other more advanced GNNs could be used in Stage 2, so this modular approach is also well adapted to future improvements in GNN algorithms.

#### Training the CNN models

We chose EfficientNet-B0 as the architecture for the cell classification task in Stage 1. The use of a compound coefficient to uniformly scale the dimensions of depth, width of the CNN architecture and resolution of the image makes EfficientNet more efficient for training. In particular, it uses about one order of magnitude fewer training parameters than comparable deep CNNs having similar performance. This allows smaller training sets and faster training [15]. From every tile in the dataset, $32\times 32$ pixel images were extracted, each containing the bounding rectangle for the centroid of an annotated cell nucleus. These $32\times 32$ pixel images were then up-scaled to $224\times 224$ pixel images using bicubic interpolation and used to train EfficientNet.

A CNN can be mathematically represented as stacked layers of non-linear function blocks applied on input data [15]. This representation can be expressed as

1 $$\begin{aligned} {\mathcal {N}} =\underset{i=1,\ldots , s}{\odot }{\mathcal {F}}_i^{L_i} (X_{\langle H_i, W_i,C_i \rangle }), \end{aligned}$$

where ${\mathcal {N}}$ represents the CNN, ${\mathcal {F}}_i$ denotes the non-linear function block at stage *i*, ${L}_i$ represents the network length and $\odot$ represents the composition of all layers stacked together. Furthermore, $\langle H_i, W_i, C_i\rangle$ represents the height, width and channel information of the input image which describes the input shape of the tensor input data to ${\mathcal {N}}$. The main goal of the EfficientNet architecture is to maximise the accuracy of the model without modifying ${\mathcal {F}}_i$. This is achieved by compound scaling of the $L_i$ (depth), $\langle H_i, W_i \rangle$ (resolution) and $C_i$ (width) of ${\mathcal {N}}$ as shown in Eq. (2) [15].

2 $$\begin{aligned}{} & {} \underset{d,w,r}{max}\ \, \;\; \text {Accuracy} ({\mathcal {N}}(d, w, r)) \end{aligned}$$

3 $$\begin{aligned}{} & {} \text {such that} \;\;\;\;\; {\mathcal {N}}(d, w, r) =\underset{i=1,\ldots , s}{\odot }{\mathcal {F}}_i^{\, d \cdot L_i} (X_{\langle \,r \cdot H_i, \, r\cdot W_i,\,w \cdot C_i \rangle }), \end{aligned}$$

where *d*, *w*, *r* are the coefficients for scaling the depth, width and resolution of ${\mathcal {N}}$. The architectural details of the EfficientNet B0 model used are given in Table 3, where MBConv is the mobile inverted bottleneck block [23].

**Table 3:** Architectural details of efficientNet B0 model for cell classification [15]

Stage	Function block	Resolution	#Channels	#Layers
*i*	${\mathcal {F}}_i$	$H_i \times W_i$	$C_i$	$L_i$
1	Conv $3\times 3$	$224\times 224$	32	1
2	MBConv1, $3\times 3$	$112\times 112$	16	1
3	MBConv6, $3\times 3$	$112\times 112$	24	2
4	MBConv6, $k5\times 5$	$56\times 56$	40	2
5	MBConv6, $k3\times 3$	$28\times 28$	80	3
6	MBConv6, $k5\times 5$	$14\times 14$	112	3
7	MBConv6, $k5\times 5$	$14\times 14$	192	4
8	MBConv6, $k3\times 3$	$7\times 7$	320	1
9	Conv $1\times 1$ & Pooling & FC	$7\times 7$	1280	1

The cell annotated dataset was divided into two disjoint subsets for training and testing based on a qualitative assessment of BM integrity and distribution in each tile. Three different data splits were used: (i) 60:40, (ii) 65:35 and (iii) 70:30 to assess the adequacy of the dataset size and to detect overfitting in the CNN model. 15% of the training data was reserved for validation in each split.

Medical image datasets are known to have class imbalance problems, where the distribution of samples representing each class is highly skewed [24]. To address this issue, oversampling of training data was performed according to cell class, to achieve a more uniform distribution of samples per class. To further artificially increase the size of the dataset, and improve CNN model generalisation on new data, the following data augmentation steps were performed on the training data. The images were flipped horizontally with a 50% probability and flipped vertically with the same probability. The complete dataset was then used to train an EfficientNet model with the objective to accurately classify each cell. No model pretraining was used.

For nuclei localisation, we used the EfficientDet-D0 architecture which integrates a previously trained EfficientNet model as its backbone network [16]. This allows transfer of the cell classification model to the nuclei localisation task. EfficientDet-D0 consists of three Bi-directional Feature Pyramid Network (BiFPN) blocks with 3x3 convolutional layers, 64 channels and the ReLU activation function. The role of BiFPN is to aggregate the features from different levels via a top-down and bottom-up approach using the convolutional layers. Table 4 shows the architectural setup of EfficientDet D0 model used in this research.

**Table 4:** Architectural details of EfficientDet D0 model for cell detection [16]

Input size	Backbone	#Channels (BiFPN)	#Layers (BiFPN)	#Layers (Box/class)
*R*_*input*_	Network	*W*_*bifpn*_	*D*_*bifpn*_	*D*_*class*_
512	B0	64	3	3

Due to the large size of the tiles ($2000 \times 2000$ pixels), each tile was split into 16 sub-tiles of size $512\times 512$ pixels, with a 16 pixel overlap between each crop to evenly distribute the squares over the image. Sub-tiles containing less than 10 cell annotations were discarded. In addition to oversampling and data augmentation techniques to handle the class imbalance problem, an $\alpha$-balanced variant of the focal loss function was used [25]. Focal loss is an improved version of the cross-entropy loss function for class imbalance in object detection tasks [26]. The focal loss function is defined by,

4 $$\begin{aligned} {{\mathcal {F}}}{{\mathcal {L}}} = {\left\{ \begin{array}{ll} - \alpha (1-p)^\gamma log(p), &{} \text {y=1} \\ - (1-\alpha )p^\gamma log(1-p), &{} \text {otherwise.} \end{array}\right. } \end{aligned}$$

Here $\alpha \in [0,1]$ is a weighting factor used to balance the positive and negative labeled samples, and $p \in [0,1]$ is the model estimated probability for class membership. The central idea of focal loss is to ignore the cases where the prediction is wrong and *p* is small, and focus more on hard-negative cases where there is a wrong prediction with a high *p* value. Here $\gamma$ is a parameter to specify the rate at which easy examples are down-weighted to focus more on the hard-negatives.

#### Training the GNN models

After constructing the node labelled cell-graph $G = (V, E, \lambda )$ as the output of Stage 1, the task of predicting the edge label values $\varepsilon (e)$ (i.e. *crossing* or *not crossing*) was considered as a GNN classification problem.

For generating a fixed dimension embedding of an arbitrary sized cell-graph *G* into ${\mathbb {R}}^n$ we use GraphSage [22]. For each target node $v \in V$, GraphSage generates an embedding of *v* by message passing, i.e. aggregating features from the local neighborhood of *v* in *G* using an iterative process. During each iteration, a fixed subset of the node’s neighborhood is sampled. All the information from the sampled neighborhood is then transformed to an embedding vector using an aggregator function. The number of graph convolutional layers K, is a hyperparameter which determines the number of hops or depth traversal, to aggregate the node information during each iteration. The aggregated information $h^k_{N_v}$ at a node $v \in V$, at the $k^{th}$ layer of a GraphSage model is defined by,

5 $$\begin{aligned} h^k_{N_v} = AGG_k (h^{k-1}_u, \forall u \in N_v) . \end{aligned}$$

Here, $h^{k-1}_u$ represents the embedding of node *u* in the previous layer $k-1$, $N_v$ represents the sampled neighborhood of node *v* and $AGG_k$ for each $k \in \{1,2,\ldots ,K\}$ is a differentiable aggregator function. The state embedding $h^k_v$ of a node *v* is calculated by concatenating the aggregated information in Eq. (5) with its state embedding in the previous layer $h^{k-1}_v$ and is defined by,

6 $$\begin{aligned} h^k_v = \sigma (W_k \cdot [ h^{k-1}_v || h^k_{N_v} ]) . \end{aligned}$$

Here, $W_k$ is a trainable weight matrix, || represents the concatenation operation and $\sigma$ represents a non-linear activation function. We used the Max Pool aggregator [22] as the aggregator function in our GraphSage model, which is defined in Eq. (7).

7 $$\begin{aligned} AGG_k^{max\_pool} = max (\{\sigma (W_{pool} h^k_{u_i}+b) : u_i \in {\mathcal {N}}(u)\}), \end{aligned}$$

where *max* is the element-wise max operator and $$\sigma $$ is a non-linear activation function. The final embedding representation $$z_v$$ of the node *v* is generated after the last iteration in the final layer K, and is defined by $$ z_v = h^k_v$$ for each $$v \in V$$.

We implemented edge label prediction from the node embedding output of GraphSage as follows. To calculate the edge information of the nodes, the node embeddings $z_v$ are fed to a fully connected neural network with output dimension 1. The result is an output vector $o_v \in {\mathbb {R}}^{ 1 \times |V|}$ where |*V*| is the number of nodes in the cell-graph. The edge label prediction between two nodes $o_{edge(i,j)} \in [0,1]$ is calculated by,

8 $$\begin{aligned} o_{edge(i,j)} = \sigma (o^T_i \cdot o_j), \end{aligned}$$

where $\sigma$ is the non-linear sigmoid function and $o_{edge(i,j)}$ is the probability that the edge between node *i* and node $j, (i,j\in V)$ crosses the BM.

**Fig. 3:**
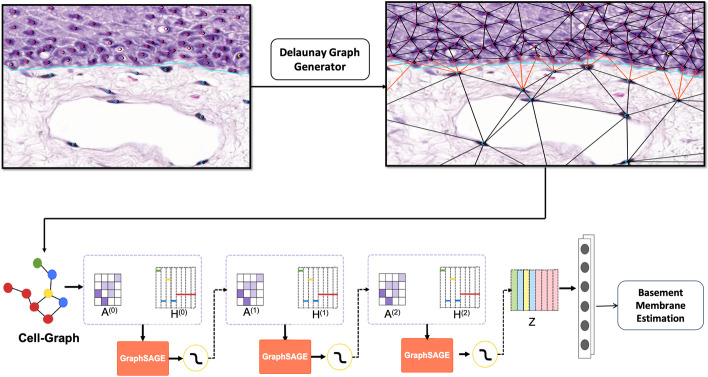
Training procedure for a GraphSage model to identify the edges crossing the BM. Ground truth annotations for nuclei locations, cell class and BM localisation were integrated with a d-distance limited Delaunay triangulation to create a node and edge labelled ground truth cell-graph. Nodes were labelled according to cell class and edges were labelled as crossing (red edges) or not crossing (black edges) the BM. Subsequently the ground truth cell-graphs were employed for training GraphSage for edge classification

Figure 3 depicts the procedure for ground truth cell-graph construction and training GraphSage to identify the edges crossing the BM. The ground truth cell-graphs for GNN training were constructed using 42 of the 51^4^ tiles from the ground truth cell annotated image set $\{ I_1 , \ldots , I_n \}$ used for CNN training. Each tile contained a visible BM annotated as a set of splines $S(I) = \{ s_1 , \ldots , s_n \}$ using the JSON format. To label each edge *e* in *G*(*I*), we computed its intersection with each of the splines $s_j \in S(I)$. If the intersection of *e* and $s_j$ was non-empty for some spline $s_j \in S(I)$ then $\varepsilon (e)$ was set to *true* otherwise $\varepsilon (e)$ was set to *false*. With the exception of the manually generated BM spline annotations *S*(*I*), construction of each cell-graph *G*(*I*) from its tissue image *I* was a fully automated process. Applied to a set of H &E stained training images $\{ I_1 , ..., I_n \}$, this process gave a ground truth cell-graph dataset $\{ G(I_1) , ..., G(I_n) \}$ of node and edge labelled cell-graphs $G(I_j)$ suitable for GNN training.

The ground truth cell-graph dataset was then used to train a GraphSage GNN. For this purpose, the cell-graph dataset was divided into two disjoint subsets for training and testing using (i) 60:40, (ii) 65:35 and (iii) 70:30 training/test data splits. Again, 15% of the training data was kept as the validation set to tune the model during training. The number of layers in the GraphSage model was set to two, as deep GNN models are susceptible to oversmoothing [27, 28]. The neighbourhood sample size was set to 10, which was adequate to completely sample the immediate neighbourhood of each node in the Delaunay triangulation. The model was trained for 100 epochs with a batch size of 32. An Adam optimizer was used with an initial learning rate set to $1\times e^{-3}$ and was scheduled to drop by $1\times e^{-1}$ after every 40 epochs. This setting allowed larger weight changes at the start of the learning process, and smaller weight changes towards the end for fine-tuning. To avoid overfitting of the data, regularisation techniques like dropout were used with a value set to 0.3 and the weight-decay parameter was set to $1\times e^{-4}$.

### Evaluation metrics

We use the standard ML metrics of accuracy, precision, recall and F1-score to evaluate the quality of trained models for both classification tasks (cell-type in the CNN and BM edge crossing in the GNN) [29]. The accuracy metric represents the number of correctly classified instances divided by the total number of instances evaluated. Precision is defined as the ratio of correctly identified positive instances to all correctly identified instances. Recall measures the proportion of correctly identified positive instances to all actual positive instances. The F1 score is the harmonic mean of precision and recall, and is a good metric for imbalanced datasets such as ours. The mathematical definitions of these metrics are given below.

9 $$\begin{aligned} Accuracy= & {} \frac{TP + TN}{TP + TN + FP+ FN} \end{aligned}$$

10 $$\begin{aligned} Precision= & {} \frac{TP}{TP+ FP} \end{aligned}$$

11 $$\begin{aligned} Recall= & {} \frac{TP}{TP+FN} \end{aligned}$$

12 $$\begin{aligned} F1= & {} \frac{2*Precision*Recall}{Precision+Recall} \end{aligned}$$

Here, *TP*, *FP*, *TN* and *FN* represent the numbers of true positives, false positives, true negatives and false negatives respectively.

To evaluate the quality of training for the nuclei localisation task, we used the mean average precision (mAP) metric which is widely used for object detection tasks [30–32]. The mAP metric is defined as the mean of the average precision (AP) for the images in a dataset, with AP defined by,

13 $$\begin{aligned} AP = \int _{0}^{1} p(r) \,dr. \end{aligned}$$

Here, *p* is the precision and *r* is the recall, $p, r \in [0,1]$. AP is thus the trade-off between precision and recall of the model.

#### Computing platform

The CNN and GNN stages of the pipeline were both implemented using the PyTorch Geometric library [33]. All training experiments were run on an NVIDIA Tesla V100 SXM2 GPU with 32GB RAM.

## Results

In this section, we discuss the quality of our CNN and GNN models in terms of their predictive performance using the above standard metrics. We also discuss their predictive performance from a histological perspective by presenting and discussing specific examples of ML labelled tiles.

We begin by summarising the overall performance of the entire ML pipeline, in Table  5, as well as the performance of its two individual ML components:

1. Accuracy and mAP of the CNN models of Stage 1,
2. Accuracy of the GNN model of Stage 2,
3. Accuracy of the entire ML pipeline, i.e. Stages 1 and 2 combined.

For each column in Table 5, we present metrics obtained for each of the three training:test data splits: (i) 60:40 (ii) 65:35 and (iii) 70:30.

**Table 5:** Comparison of ML performance on validation (Val) and test datasets

Splits	EfficientNet		EfficientDet	GraphSage		Entire ML pipeline
	Stage 1		Stage 1	Stage 2		Stages 1 + 2
	**Accuracy**		**mAP**	**Accuracy**		**Accuracy**
	Val	Test	Test	Val	Test	Test
60:40	**0.9811**	0.7103	0.5181	0.9123	0.9041	0.8781
65:35	0.9801	0.6985	**0.5250**	0.9016	0.8970	0.8669
70:30	0.9808	**0.7115**	0.5095	**0.9148**	**0.9071**	**0.8816**

Best outcomes in bold

### CNN evaluation

#### Cell classification

We computed values for the standard metrics of precision, recall, F1-score and accuracy to understand the results of training an EfficientNet CNN for the cell classification task. The detailed metrics for the validation and test datasets are shown in Table 6 and Additional file 1: Tables S2 and S3. The best result was obtained for the 70:30 split experiment, where F1 scores for the fibroblast and endothelial, and epithelial classes are high. These cell classes predominate on opposite sides of the BM and help locate the BM, especially when intact. However, the high precision, recall and F1 scores for inflammatory cells with respect to the validation set coupled with low corresponding values for the test set suggest partial overfitting of the CNN model due to a lack of sufficient inflammatory cell examples.

**Table 6:** Detailed metrics for cell class identification—(70:30 split)

	Precision	Recall	F1	Support
EfficientNet (Stage 1)—Validation Set				
* Inflammatory*	0.99	1.00	1.00	2534
* Lymphocyte*	0.97	1.00	0.99	7036
* Fibroblast/Endothelial*	0.98	0.97	0.97	6739
* Epithelial*	0.98	0.97	0.97	4286
* Accuracy*			**0.98**	20595
EfficientNet (Stage 1)—Test Set				
* Inflammatory*	0.06	0.05	0.05	401
* Lymphocyte*	0.68	0.33	0.45	4479
* Fibroblast/Endothelial*	0.62	0.79	0.69	6433
* Epithelial*	0.83	0.90	0.86	7882
* Accuracy*			**0.71**	19195

Accuracy measurements in bold

#### Nuclei localisation

We evaluated the performance of EfficientDet on the nuclei localisation task using the mAP metric. We recorded the best mAP value for the 65:35 dataset split ratio and the localisation performance by cell class for the test dataset is shown in Table 7 (and Additional file 1: Tables S4 and S5). Recall that EfficientDet builds upon an EfficientNet model. Comparison of Tables 6 and 7 illustrates how this dependency affects the accuracy of localising cell nuclei.

However, the mAP metric does not give a representative measure of localisation performance when the training set is not fully annotated. It is not practical to fully manually annotate all ground truth images, especially for new datasets. This meant there were many cases in which the ML model accurately located a cell nucleus which was not annotated in the ground truth image. In such a case, according to mAP the prediction was considered false, which is incorrect.

Verification of both cell class predictions and locations was carried out manually through visual inspection by the histopathology experts (Additional file 1: Fig. S1).

**Table 7:** Detailed metrics for nuclei localisation—65:35 split

Inflammatory	Lymphocyte	Fibroblast/endothelial	Epithelial	
EfficientDet (Stage 1)—Test Set				
mAP	mAP	mAP	mAP	*Average mAP*
0.0881	0.5105	0.6466	0.8546	**0.5250**

Average mAP in bold

### GNN evaluation

The GraphSage GNN model generates predictions of edge labels in the cell-graph which identify those edges that cross the BM. Table 8 (and Additional file 1: Tables S6 and S7) presents a summary of GNN model performance. For the ground-truth cell-graph dataset, we observed the best result for the 70:30 split experiment with a good F1 score of 0.9071. Notice that the class imbalance in the ground truth cell-graph training set (see Table 2) manifests as an F1 value imbalance between crossing and non-crossing edges. However, the training set imbalance has been ameliorated, and the overall F1 score is good.

**Table 8:** Detailed metrics for edge classification—70:30 split

	Precision	Recall	F1	Support
GraphSage (STage 2)—validation set				
* Non-crossing edge*	0.9795	0.9293	0.9537	20017
* Crossing edge*	0.3552	0.6672	0.4636	1169
* Accuracy*			**0.9148**	21186
GraphSage (Stage 2)—test set				
* Non-crossing edge*	0.9810	0.9194	0.9492	45779
* Crossing edge*	0.3417	0.7011	0.4594	2733
* Accuracy*			**0.9071**	48512

Accuracy measurements in bold

### Full pipeline evaluation

The goal of metrics for the full ML pipeline was to estimate its performance at predicting the BM in original and unannotated images of H &E stained oral mucosa tissue, imitating clinical usage. This need not be the same as measured GNN performance on the cell-graph test data described in section "Methods". This is because the overall accuracy of the pipeline depends on the accuracy of two different ML models in Stages 1 and 2. These have been shown to be less than 100% accurate (Tables 5, 6, 7, and 8). To evaluate the performance metrics, it was necessary to add BM ground truth annotations to the digital images as spline data.

**Table 9:** Detailed metrics for the full pipeline—70:30 split

	Precision	Recall	F1	Support
Full pipeline (stages 1 + 2)—test set				
* Non-crossing edge*	0.9794	0.8944	0.9350	55629
* Crossing edge*	0.2318	0.6288	0.3387	2818
* Accuracy*			**0.8816**	58447

Accuracy measurement in bold

**Fig. 4:**
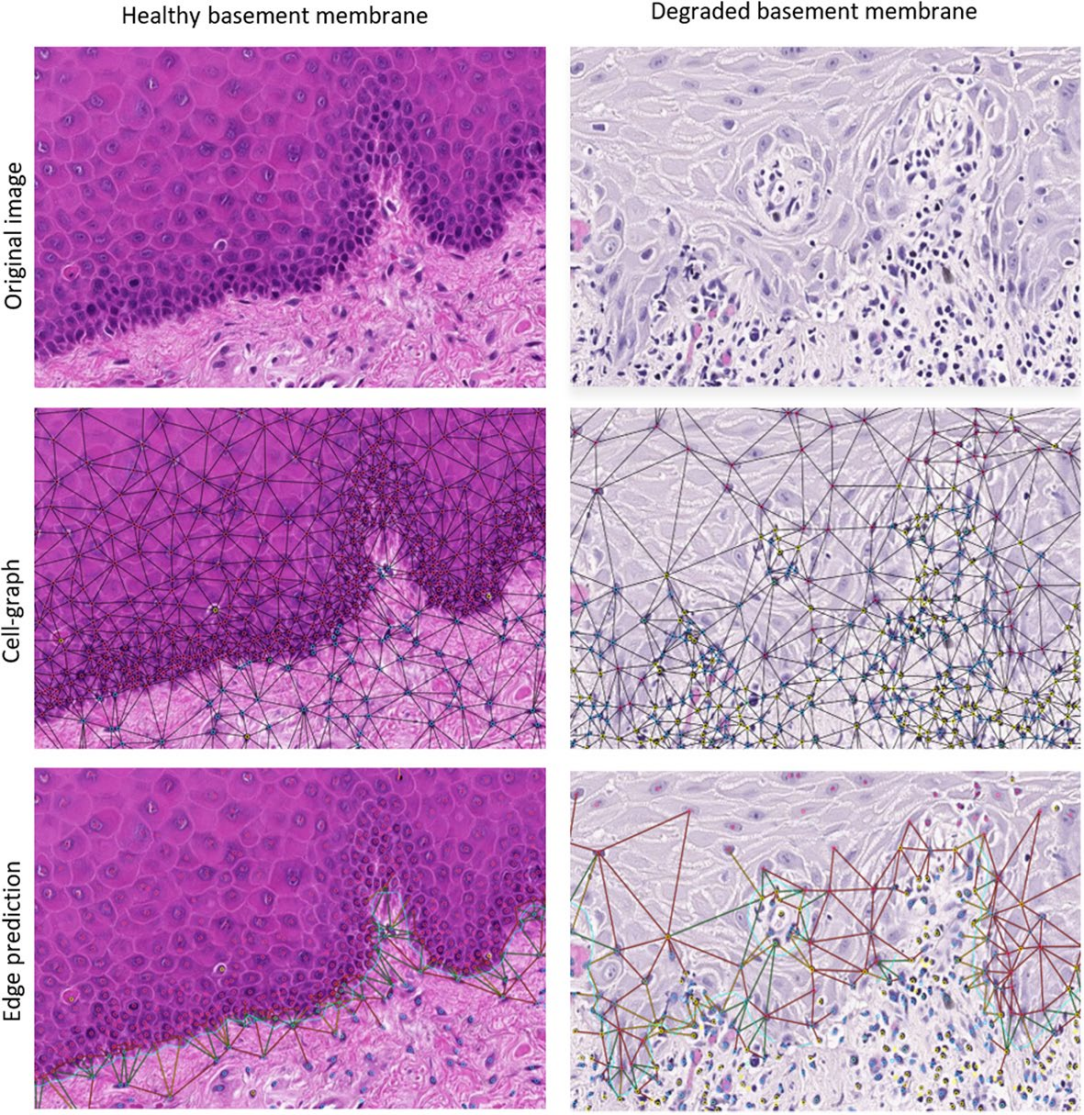
A comparison of BM crossing edge predictions by the pipeline in tissue displaying a healthy BM and in tissue with severe degradation of the BM. In the bottom row, predicted edge labels (crossing/not crossing the BM) are visualised as true positive (green), false negative (yellow) and false positive (red). True negative edges are not visualised. The ground truth annotation of the BM is superimposed as a blue line for comparison

The observed performance results given in Table 9 show only a small drop from 0.91 to 0.88 in the F1 metric in comparison to Table 8 (and Additional file 1: Tables S8 and S9).

These metric estimations were visually confirmed by histology experts. The predicted BM crossing edges were confirmed to follow the BM interface with good agreement. Figure 4 highlights two cases of healthy and severely inflamed tissue. In healthy tissue and intact BM, the pipeline performed well according to visual inspection. Whereas with increasing inflammation, witnessed by exocytosis and elevated numbers of inflammatory and lymphocytic cells, resulting in loss of BM integrity, the false positive edge predictions increase according to visual inspection.

## Discussion

We have presented a two-stage ML pipeline, based on a CNN and a GNN architecture, to identify and localise low-level and higher-level histological features as node and edge labels in cell-graphs. This pipeline achieves good levels of accuracy using currently available ML technology.

Graph-based learning in computational pathology includes a variety of tasks, applications, datasets and ML architectures, which have been combined in different ways. Additional file 1: Table S1 provides a representative overview of existing research organised in terms of both ML techniques and digital pathology goals. The table identifies five clusters of published research (Groups I to V) and ranks these in order of similarity to the current study, Group GI being the most and Group V the least similar. Our work aimed to develop an ML architecture to identify high-level histological features from cell-graphs. Thus it shares a common goal with Groups I and II which is not shared with the larger Groups III, IV and V (c.f. Additional file 1: Table S1 column 10).

In Group I, Levy [34] and Anklin [35] are the two works most closely related to our approach overall. In common with our work, they share: (i) a DP goal of predicting high-level histological features from a graph and (ii) an ML approach of using GNN algorithms. In Levy [34] a framework is introduced for capturing the micro- and macro-architectures of histological images to determine the degree of tumor invasion. Both CNN and GNN algorithms were used to learn high-level histology features as 2-dimensional regions of interest, which are collections of tissue patches. For example, the degree of overlap between the tumor and adjacent tissue region can thus be measured. By contrast, the BM, which we study, is essentially a 1-dimensional structure, consisting of highly specialised connective tissue, not easily representable as a region. The work of Anklin [35] utilises the tissue-graphs introduced by Pati [36] for hierarchical cell-graphs to propose a weakly supervised GNN for segmentation into regions of benign or diseased tissue that are biologically meaningful to a pathologist.

Both Levy [34] and Anklin [35] use tissue regions/patches as nodes in their cell-graph representation. Such composite node models risk being too large to capture individual cell interactions. By contrast, we use individual cell nuclei as the nodes in our cell-graphs, since our aim is to study the cellular interactions across the BM. This observation can also be applied to the research in Group V of Additional file 1: Table S1.

The work of Levy [34] comes closest to replicating our combined CNN/GNN ML architecture for histological feature identification using supervised learning. However, edge features are not used in either Levy [34] or Anklin [35]. Thus, segmentation by edge classification could not be implemented by either of these approaches. Our approach integrates edge label aggregation with node label aggregation to extend an existing message passing GNN (GraphSage) to edge label prediction. This combined aggregation technique could also be applied to other message passing GNNs, which are increasingly the dominant technology of GNNs (see for example [37]). Our work highlights the fact that optimal aggregation of node and edge data is an important area for future cell-graph research. The work presented here thus represents a novel approach to segmentation problems using edge label prediction.

Additional file 1: Table S1 also shows that Levy [34] and Anklin [35] differ in the specific CNN and GNN algorithms used, as well as the graph construction method. Furthermore, Anklin [35] uses a semi-supervised approach to learning in contrast with the supervised approach used both here and in Levy [34].

Learning high level histological features from graphs by deep learning methods other than GNNs has also been proposed. Three such examples are given in Additional file 1: Table S1 Group II. The work of Sirinukunwattana [8] introduced a cell-graph approach to extract the tissue phenotype signatures using statistical measurements and logistical regression analyses. The study involved clustering of tissue patches, which was further employed as a method for tissue phenotyping [9, 38]. These papers share some similarities with our paper, as they also use supervised CNN algorithms for cell identification and detection [8]. Furthermore, they also use the Delaunay triangulation method for their cell-graph construction. However, in our approach we use EfficientNet and EfficientDet [15, 16] for cell identification and detection, which are considered state-of-the-art methods in the CNN community. Also, we use GNNs instead of clustering or logistic regression analyses for understanding the high-level histopathological features.

Many works consider a combination of CNN and GNN learning algorithms to predict an overall classification for an entire cell-graph such as disease severity. All these works contrast with our own goal of classifying the individual graph components and substructures. To survey this extensive research, it has been divided into three categories (Groups III, IV and V) in Additional file 1: Table S1.

The majority of the studies in the field of digital pathology involving cell-graphs or tissue-graphs perform ML only from node features (Group III: [39–41]). This shows that the use of edge labels as topology descriptors for biological entity-graphs is under-exploited. To our knowledge Sureka [39], Anand [40], and Studer [41], as well as Bilgin [10] in Group IV are the only works which utilise an edge attribute for learning, namely the distance between nodes. The edge convolution method proposed by Anand [40] was used in Sureka [39] to incorporate the edge distance attribute. The ENN network [42] that maps edge features to a matrix that is multiplied by the node feature vector, was used in Studer [41].

The research works in Group IV [11, 12, 43–45] also combine CNN and GNN methods to classify overall graph properties, and all share a common use of the k-nearest neighbour (kNN) method for cell-graph construction, and cell nuclei as the node attributes. By contrast, our approach uses Delaunay triangulation for cell-graph construction. We use the Delaunay method because kNN-based edge generation is not invariant with respect to the node ordering in a graph. By contrast, both the Delaunay triangulation and *d*-distance neighbors methods used in our approach are invariant to node order [46]. The important consequence when using kNN edge generation, is that non-isomorphic^5^ cell-graphs can be generated from the same tissue image. Consequently, kNN methods cannot be guaranteed to be robust to tissue orientation, which can negatively impact the learnability of histological features. However, in Group IV, kNN methods are not applied to learn histological features, so kNN methods may nevertheless be robust for learning the tasks of this group.

The research works in Group V [10, 36, 47, 48] all make use of complex node representations in cell-graphs, such as cell-graphs, cell clusters, patch graphs and tissue-graphs. This also holds for both the works of Levy [34] and Anklin [35] in Group I. Hierarchical graphs have also been used to model the tissue structure. The work of Pati [36] proposed HACT graphs, which are a combination of cell-graph, tissue-graph and cell-to-tissue-graphs for breast cancer classification. The work of Lu [47] generated cell cluster graphs using the Mean-shift clustering algorithm by grouping nuclei into subgraphs based on their spatial and morphological features. In Bilgin [10] and Demir [48] hierarchical cell cluster graphs were constructed by placing a grid on top of the cells and clustering the cells based on the grid size and number of cells within it. The cell cluster generated by this method can represent a single cell, part of a cell or a group of cells combined.

As for Group I, we can again observe that composite node models risk being too large to capture individual cell interactions. By contrast, we use cell nuclei as the nodes in our cell-graphs, since our aim was to learn and represent the cellular interaction across the BM. For this reason, we consider graph representations other than cell-graphs to be largely outside the scope of our work.

There are several limitations to this study. We observe that even though CNN model performance was not optimal for all four cell types due to data imbalances, the GNN model was robust to CNN prediction errors, and actually achieved greater accuracy (GNN overall accuracy 0.91 versus CNN overall accuracy 0.71). Data imbalances were due to the inherent nature of the dataset with a combination of healthy and diseased tissues. Thus, additional research is warranted to address the data imbalances in CNN training data. Future algorithmic research might also assess whether additional GNN stages in our pipeline are effective in constructing hierarchical cell-graph models [10, 36]. The node and edge aggregation methods used here could potentially be improved by further algorithmic analysis of GNNs together with practical ML experimentation. Additional classes of histologically-based edge labels could also be considered to further improve accuracy.

## Conclusions

We have shown that convolutional and graph neural networks are complementary technologies for learning, representing and predicting local and global histological features using node and edge labels from digitised tissue images. CNNs are well suited to local analysis of small image regions. They yield low-level histological representations that can be passed on for higher-level topological analysis by GNNs, through the shared data structure of a cell-graph model. This process is naturally hierarchical, and follows the workflow of the pathologist in clinical practise. A staged CNN/GNN pipeline also enhances the explainability of ML predictions in histological terms terms that can be easily understood by the clinician, especially when predicted features are linked to a diagnostic model, such as oral mucosal cGvHD [13]. Using state-of-the-art but off-the-shelf CNN and GNN technologies, we have demonstrated good performance results for such a staged ML pipeline. In the future, these performance results could be improved by using larger and more balanced data sets for ML training, and further improvements in CNN and GNN algorithms. By using precisely defined cell-graph structures for data exchange between ML models, a staged ML pipeline is also robust to future changes in ML technology, and developments in diagnostic techniques.

The work presented here is oriented towards oral medicine, but we believe the approach is general, and could be applied to other medical fields such as oncology.

## Supplementary Information

**Additional file 1**. Supplementary Figure 1 and Tables 1 to 9.

## Data Availability

The data is not publically available due to privacy. The datasets used and/or analysed during the current study are available from the corresponding author on reasonable request.

## Footnotes

1 Topological properties are geometry invariant, and hence less sensitive to problems such as tissue damage and staining distortion which can occur in the digital imaging process for tissue samples.

2 Note that for representing an undirected edge, the node order is considered irrelevant, i.e. $\{v_1, v_2\}$ is the same as $\{v_2, v_1\}$.

3 For *d* we have chosen a value effectively equivalent to 300 pixels (150 microns). This choice was made to avoid long edges in the cell-graph, as cell density is generally not the same in different parts of a tile.

4 Absence of BM was the excluding factor.

5 Observe that most important graph theoretic concepts (e.g. cliques, centroids) are isomorphism invariant.

## References

[1] Srinidhi CL, Ciga O, Martel AL (2021). Deep neural network models for computational histopathology: a survey. Med Image Anal.

[2] Ahmedt-Aristizabal D, Armin MA, Denman S, Fookes C, Petersson L. A survey on graph-based deep learning for computational histopathology. Comput Med Imaging Graphics. 2021;102027. 10.1016/j.compmedimag.2021.102027 34959100

[3] Scarselli F, Gori M, Tsoi AC, Hagenbuchner M, Monfardini G (2008). The graph neural network model. IEEE Trans Neural Netw.

[4] Sowa JF, editor. Principles of semantic networks - explorations in the representation of knowledge. San Mateo, California: The Morgan Kaufmann Series in representation and reasoning. Morgan Kaufmann; 1991.

[5] Aggarwal CC, Wang H. Graph data management and mining: A survey of algorithms and applications. In: Managing and Mining Graph Data, pp. 13–68. Springer, Boston, MA; 2010.

[6] Needham M, Hodler AE (2019). Graph algorithms: practical examples in apache spark and Neo4j.

[7] Gunduz C, Yener B, Gultekin SH. The cell graphs of cancer. Bioinformatics. (suppl_1)2004;20:145–51. 10.1093/bioinformatics/bth933 15262793

[8] Sirinukunwattana K, Snead D, Epstein D, Aftab Z, Mujeeb I, Tsang YW, Cree I, Rajpoot N (2018). Novel digital signatures of tissue phenotypes for predicting distant metastasis in colorectal cancer. Sci Rep.

[9] Javed S, Mahmood A, Fraz MM, Koohbanani NA, Benes K, Tsang Y-W, Hewitt K, Epstein D, Snead D, Rajpoot N (2020). Cellular community detection for tissue phenotyping in colorectal cancer histology images. Med Image Anal.

[10] Bilgin C, Demir C, Nagi C, Yener B. Cell-graph mining for breast tissue modeling and classification. In: 2007 29th Annual international conference of the IEEE Engineering in Medicine and Biology Society, pp. 5311–5314;2007. IEEE 10.1109/IEMBS.2007.4353540 18003206

[11] Jaume G, Pati P, Foncubierta-Rodriguez A, Feroce F, Scognamiglio G, Anniciello AM, Thiran J-P, Goksel O, Gabrani M. Towards explainable graph representations in digital pathology; 2020. arXiv preprint arXiv:2007.00311.

[12] Jaume G, Pati P, Bozorgtabar B, Foncubierta A, Anniciello AM, Feroce F, Rau T, Thiran J-P, Gabrani M, Goksel O. Quantifying explainers of graph neural networks in computational pathology. In: Proceedings of the IEEE/CVF conference on computer vision and pattern recognition, pp. 8106–8116;2021.

[13] Tollemar V, Tudzarovski N, Warfvinge G, Yarom N, Remberger M, Heymann R, Legert KG, Sugars RV (2020). Histopathological grading of oral mucosal chronic graft-versus-host disease: large cohort analysis. Biol Blood Marrow Transpl..

[14] Kalluri R, Weinberg RA (2009). The basics of epithelial-mesenchymal transition. J Clin Investig.

[15] Tan M, Le Q. Efficientnet: rethinking model scaling for convolutional neural networks. In: International conference on machine learning, pp. 6105–6114;2019.

[16] Tan M, Pang R, Le QV. Efficientdet: scalable and efficient object detection. In: Proceedings of the IEEE/CVF conference on computer vision and pattern recognition (CVPR) 2020.

[17] Delaunay B. Sur la sphere vide. Izv. Akad. Nauk SSSR, Otdelenie Matematicheskii i Estestvennyka Nauk. 1934(793-800);7:1–2.

[18] Guibas L, Stolfi J (1985). Primitives for the manipulation of general subdivisions and the computation of Voronoi. ACM Trans Graphics.

[19] Abadal S, Jain A, Guirado R, López-Alonso J, Alarcón E (2021). Computing graph neural networks: a survey from algorithms to accelerators. ACM Comput Surv.

[20] Wu Z, Pan S, Chen F, Long G, Zhang C, Philip SY (2020). A comprehensive survey on graph neural networks. IEEE Trans Neural Netw Learn Syst.

[21] Zhou J, Cui G, Hu S, Zhang Z, Yang C, Liu Z, Wang L, Li C, Sun M (2020). Graph neural networks: a review of methods and applications. AI Open.

[22] Hamilton WL, Ying R, Leskovec J. Inductive representation learning on large graphs. In: Proceedings of the 31st international conference on neural information processing systems, pp. 1025–1035;2017.

[23] Sandler M, Howard A, Zhu M, Zhmoginov A, Chen L-C. Mobilenetv2: Inverted residuals and linear bottlenecks. In: Proceedings of the IEEE conference on computer vision and pattern recognition, pp. 4510–4520;2018.

[24] Zhang C. Medical image classification under class imbalance. Ph.D. thesis, Iowa State University;2019.

[25] Lin T-Y, Goyal P, Girshick R, He K, Dollár P. Focal loss for dense object detection. In: Proceedings of the IEEE international conference on computer vision, pp. 2980–2988;2017.

[26] Oksuz K, Cam BC, Kalkan S, Akbas E. Imbalance problems in object detection: a review. IEEE Trans Pattern Anal Mach Intell. 2020. 10.1109/TPAMI.2020.2981890 32191882

[27] Chen D, Lin Y, Li W, Li P, Zhou J, Sun X. Measuring and relieving the over-smoothing problem for graph neural networks from the topological view. In: Proceedings of the AAAI conference on artificial intelligence, vol. 34, pp. 3438–3445;2020.

[28] Li Q, Han Z, Wu X-M. Deeper insights into graph convolutional networks for semi-supervised learning. In: Thirty-second AAAI conference on artificial intelligence; 2018.

[29] Naser M, Alavi A. Insights into performance fitness and error metrics for machine learning. 2020; arXiv preprint arXiv:2006.00887.

[30] Everingham M, Van Gool L, Williams CK, Winn J, Zisserman A (2010). The pascal visual object classes (VOC) challenge. Int J Comput Vis..

[31] Everingham M, Eslami SA, Van Gool L, Williams CK, Winn J, Zisserman A (2015). The pascal visual object classes challenge: a retrospective. Int J Comput Vis..

[32] Russakovsky O, Deng J, Su H, Krause J, Satheesh S, Ma S, Huang Z, Karpathy A, Khosla A, Bernstein M (2015). Imagenet large scale visual recognition challenge. Int J Comput Vis..

[33] Fey M, Lenssen JE. Fast graph representation learning with pytorch geometric; 2019. arXiv preprint arXiv:1903.02428.

[34] Levy J, Haudenschild C, Barwick C, Christensen B, Vaickus L. Topological feature extraction and visualization of whole slide images using graph neural networks. In: BIOCOMPUTING 2021: proceedings of the pacific symposium, pp. 285–296; 2020. World Scientific. PMC7959046 33691025

[35] Anklin V, Pati P, Jaume G, Bozorgtabar B, Foncubierta-Rodriguez A, Thiran J-P, Sibony M, Gabrani M, Goksel O. Learning whole-slide segmentation from inexact and incomplete labels using tissue graphs. In: International conference on medical image computing and computer-assisted intervention, pp. 636–646; 2021. Springer.

[36] Pati P, Jaume G, Foncubierta-Rodríguez A, Feroce F, Anniciello AM, Scognamiglio G, Brancati N, Fiche M, Dubruc E, Riccio D (2022). Hierarchical graph representations in digital pathology. Med Image Anal.

[37] Nikolentzos G, Dasoulas G, Vazirgiannis M. Permute me softly: learning soft permutations for graph representations. IEEE Trans Pattern Analy Mach Intell., 1–12;2022. 10.1109/TPAMI.2022.3188911. 10.1109/TPAMI.2022.3188911 35793300

[38] Javed S, Mahmood A, Werghi N, Benes K, Rajpoot N (2020). Multiplex cellular communities in multi-gigapixel colorectal cancer histology images for tissue phenotyping. IEEE Trans Image Process.

[39] Sureka M, Patil A, Anand D, Sethi A. Visualization for histopathology images using graph convolutional neural networks. In: 2020 IEEE 20th international conference on bioinformatics and bioengineering (BIBE), pp. 331–335;2020. IEEE.

[40] Anand D, Gadiya S, Sethi A. Histographs: graphs in histopathology. In: Medical Imaging 2020: Digital Pathology, vol. 11320, p. 113200;2020. International Society for Optics and Photonics.

[41] Studer L, Wallau J, Dawson H, Zlobec I, Fischer A. Classification of intestinal gland cell-graphs using graph neural networks. In: 2020 25th International conference on pattern recognition (ICPR), pp. 3636–3643;2021. IEEE.

[42] Gilmer J, Schoenholz SS, Riley PF, Vinyals O, Dahl GE. Neural message passing for quantum chemistry. In: Precup, D., Teh, Y.W. (eds.) Proceedings of the 34th international conference on machine learning. Proceedings of machine learning research, vol. 70, pp. 1263–1272. Breckenridge, Colorado, USA 2017. https://proceedings.mlr.press/v70/gilmer17a.html.

[43] Gao Z, Lu Z, Wang J, Ying S, Shi J (2022). A convolutional neural network and graph convolutional network based framework for classification of breast histopathological images. IEEE J Biomed Health Inform.

[44] Wang J, Chen RJ, Lu MY, Baras A, Mahmood F. Weakly supervised prostate TMA classification via graph convolutional networks. In: 2020 IEEE 17th International Symposium on Biomedical Imaging (ISBI), pp. 239–243 2020. IEEE

[45] Zhou Y, Graham S, Alemi Koohbanani N, Shaban M, Heng P-A, Rajpoot N. Cgc-net: cell graph convolutional network for grading of colorectal cancer histology images. In: Proceedings of the IEEE/CVF international conference on computer vision workshops, pp. 0–0;2019.

[46] Okabe A, Boots B, Sugihara K, Chiu SN. Spatial tessellations: concepts and applications of Voronoi diagrams. 2nd ed. USA: Series in Probability and Statistics. John Wiley and Sons Inc; 2000.

[47] Lu W, Toss M, Dawood M, Rakha E, Rajpoot N, Minhas F (2022). Slidegraph+: whole slide image level graphs to predict her2 status in breast cancer. Med Image Anal.

[48] Demir C, Gultekin SH, Yener B (2005). Learning the topological properties of brain tumors. IEEE/ACM Trans Comput Biol Bioinform..

