# Supplementary material for: A graph neural network framework for mapping histological topology in oral mucosal tissue
Source: BMC Bioinformatics. 2022 Nov 25;23:506. doi: 10.1186/s12859-022-05063-5 (PMC9700957; doi:10.1186/s12859-022-05063-5)
Supplement: Supplementary file 1 — Additional file 1. Supplementary Figure 1 and Tables 1 to 9. [file 12859_2022_5063_MOESM1_ESM.pdf]

SUPPLEMENTARY MATERIAL

A Graph Neural Network Framework for Mapping Histological Topology in Oral Mucosal Tissue

Aravind Nair[1†], Helena Arvidsson[2†], Jorge Gatica[1], Nikolce Tudzarovski[2], Karl Meinke[1] and Rachael. V Sugars[2*]

*Correspondence: rachael.sugars@ki.se
[2]Division of Oral Diagnostics and Rehabilitation, Department of Dental Medicine, Karolinska Institutet, Stockholm, Sweden
†Equal contributor

Author details
[1]Division of Theoretical Computer Science, Department of Computer Science, KTH Royal Institute of Technology, Stockholm, Sweden. [2]Division of Oral Diagnostics and Rehabilitation, Department of Dental Medicine, Karolinska Institutet, Stockholm, Sweden.

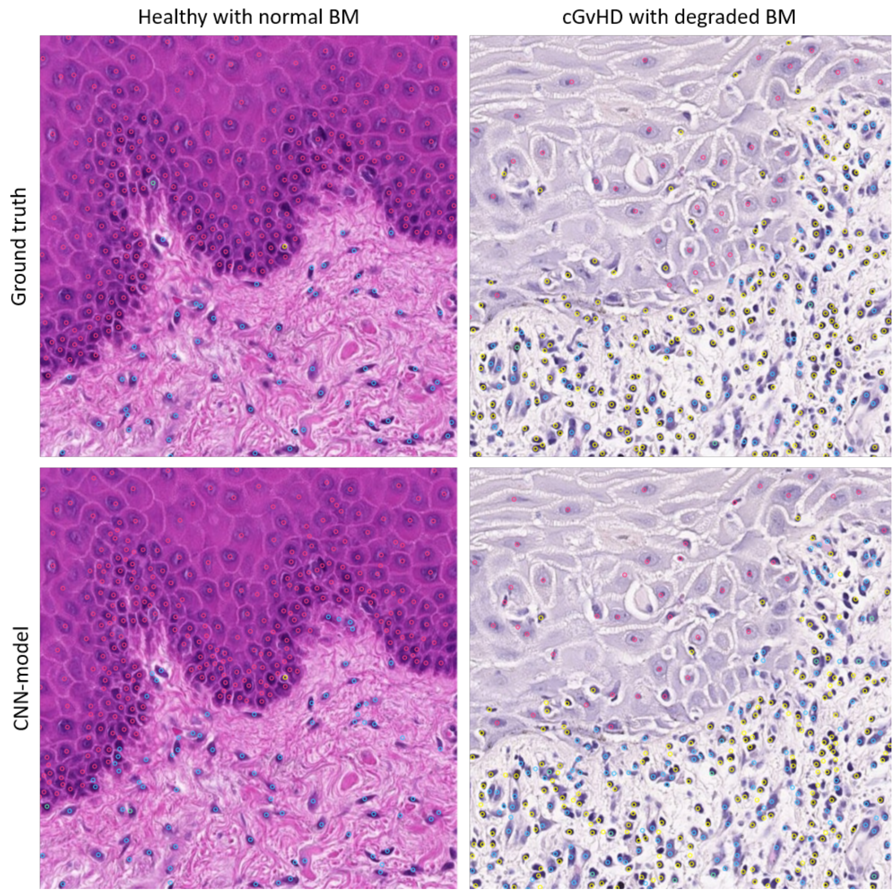

Supplementary Figure 1 Visual assessment of ground truth annotations alongside predictions from the CNN-model (70:30 split) in healthy tissue and oral mucosal cGvHD. Oral mucosal cGVHD tissue presents with inflammation, atrophy and BM degeneration. The model predictions show good agreement with ground truth. Epithelial - red, fibroblasts and endothelial – blue, inflammatory cells – green and lymphocytes - yellow.

Supplementary Table 1 Related Literature Summary provides a representative overview of existing research organised in terms of both ML techniques and digital pathology goals. The table identifies five clusters of published research (groups G0 to GV) and ranks these in order of similarity to the current study, GI being the most and GV the least similar.

Group	Article	DP Task	CNN	GNN	Graph Construction Method	Node Model	Node Labels	Edge Labels	Histology Learned from Graph
G 0	Nair et al. (this report)	Edge Classification (*Supervised*)	Yes	Yes	Delaunay	Nuclei	One hot embedding	0/1 class	Yes
G I	Levy et al [34]	Tissue Scoring (*Supervised*)	Yes	Yes	kNN	Tissue ROI	CNN Features	-	Yes
	Anklin et al. [35]	Tissue Segmentation (*Weakly Supervised*)	Yes	Yes	kNN	Tissue ROI	CNN Features	-	Yes
G II	Javed et al. [9]	Community Detection (*Semi Supervised*)	Yes	-	Delaunay	Nuclei	Hand Crafted	-	Yes
	Javed et al. [38]	Community Detection (*Unsupervised*)	Yes	-	Delaunay	Nuclei	Hand Crafted + CNN Features	-	Yes
	Sirinukunwattana et al. [8]	Tissue Phenotype (*Unsupervised*)	Yes	-	Distance	Nuclei	Hand Crafted + CNN Features	-	Yes
G III	Sureka et al. [39]	Graph Classification (*Supervised*)	Yes	Yes	Distance	Nuclei	Not Mentioned	Distance	No
	Anand et al. [40]	Graph Classification (*Supervised*)	Yes	Yes	Distance	Nuclei	Hand Crafted + CNN Features	Distance	No
	Studer et al. [41]	Graph Classification (*Supervised*)	Yes	Yes	kNN	Nuclei	Hand Crafted	Distance	No
G IV	Gao et al. [43]	Graph Classification (*Supervised*)	Yes	Yes	kNN	Nuclei	CNN Features	-	No
	Wang et al. [44]	Graph Classification (*Weakly Supervised*)	Yes	Yes	kNN	Nuclei	CNN Features	-	No
	Jaume et al. [11]	Graph Classification (*Supervised*)	Yes	Yes	kNN	Nuclei	Hand Crafted	-	No
	Jaume et al. [12]	Graph Classification (*Supervised*)	Yes	Yes	kNN	Nuclei	Hand Crafted + CNN Features	-	No
	Zhou et al.[45]	Graph Classification (*Supervised*)	Yes	Yes	kNN	Nuclei	Hand Crafted	-	No
G V	Pati et al. [36]	Graph Classification (*Supervised*)	Yes	Yes	kNN	Nuclei, Tissue ROI	CNN Features	-	No
	Lu et al. [47]	Graph Classification (*Weakly Supervised*)	Yes	Yes	Delaunay	Cell Clusters	Hand Crafted + CNN Features	-	No
	Bilgin et al. [10]	Graph Classification (*Supervised*)	No	No	Delaunay	Nuclei, Cell Clusters	Hand Crafted	Weighted	No
	Demir et al.[48]	Graph Classification (*Supervised*)	No	No	Complete Graph	Cell Clusters	Hand Crafted	Distance	No
G VI	Gunduz et al. [7]	Graph Classification (*Supervised*)	No	No	Waxman Model	Nuclei	Hand Crafted	-	No

Supplementary Table 2 Detailed metrics for cell class identification - (60:40 split)

**EfficientNet (Stage 1) - Validation Set**				
	**Precision**	**Recall**	**F1**	*Support*
Inflammatory	1.00	1.00	1.00	2068
Lymphocyte	0.97	1.00	0.99	5458
Fibroblast/Endothelial	0.98	0.98	0.98	5163
Epithelial	0.99	0.95	0.97	3578
**Accuracy**			**0.98**	16267
**EfficientNet (Stage 1) - Test Set**				
Inflammatory	0.09	0.05	0.06	542
Lymphocyte	0.54	0.30	0.39	4097
Fibroblast/Endothelial	0.62	0.81	0.70	8062
Epithelial	0.87	0.83	0.85	10237
**Accuracy**			**0.71**	22938

Supplementary Table 3 Detailed metrics for cell class identification - (65:35 split)

**EfficientNet (Stage 1) - Validation Set**				
	**Precision**	**Recall**	**F1**	*Support*
Inflammatory	1.00	1.00	1.00	2301
Lymphocyte	0.98	1.00	0.99	6537
Fibroblast/Endothelial	0.98	0.97	0.97	6076
Epithelial	0.98	0.95	0.97	3929
**Accuracy**			**0.98**	18843
**EfficientNet (Stage 1) - Test Set**				
Inflammatory	0.10	0.04	0.06	531
Lymphocyte	0.63	0.29	0.40	4978
Fibroblast/Endothelial	0.61	0.80	0.69	8091
Epithelial	0.82	0.86	0.84	9661
**Accuracy**			**0.70**	23261

Supplementary Table 4 Detailed metrics for nuclei localisation - 60:40 split

**EfficientDet (Stage 1) - Test Set**				
Inflammatory mAP	*Lymphocyte* mAP	*Fibroblast/Endothelial* mAP	*Epithelial* mAP	*Average mAP*
0.0782	0.5310	0.6076	0.8554	**0.5181**

Supplementary Table 5 Detailed metrics for nuclei localisation - 70:30 split

**EfficientDet (Stage 1) - Test Set**				
Inflammatory mAP	*Lymphocyte* mAP	*Fibroblast/Endothelial* mAP	*Epithelial* mAP	*Average mAP*
0.0415	0.5007	0.6454	0.8505	**0.5095**

Supplementary Table 6 Metrics for edge classification on ground-truth validation and test datasets - 60:40 split

**GraphSage (Stage 2) - Validation Set**				
	**Precision**	**Recall**	**F1**	*Support*
Non-Crossing Edge	0.9821	0.9240	0.9522	20017
Crossing Edge	0.3533	0.7109	0.4720	1169
**Accuracy**			**0.9103**	21186
**GraphSage (Stage 2) - Test Set**				
Non-Crossing Edge	0.9811	0.9156	0.9472	59562
Crossing Edge	0.3526	0.7231	0.4740	3785
**Accuracy**			**0.9041**	63347

Supplementary Table 7 Metrics for edge classification on ground-truth validation and test datasets - 65:35 split

**GraphSage (Stage 2) - Validation Set**				
	**Precision**	**Recall**	**F1**	*Support*
Non-Crossing Edge	0.9839	0.9107	0.9459	20017
Crossing Edge	0.3277	0.7451	0.4552	1169
**Accuracy**			**0.9016**	21186
**GraphSage (Stage 2) - Test Set**				
Non-Crossing Edge	0.9835	0.9061	0.9432	55944
Crossing Edge	0.3228	0.7461	0.4506	3356
**Accuracy**			**0.8970**	59300

Supplementary Table 8 Metrics for the full pipeline - 60:40 split

**Full Pipeline (Stages 1 + 2) - Test Set**				
	**Precision**	**Recall**	**F1**	*Support*
Non-Crossing Edge	0.9779	0.8919	0.9329	89061
Crossing Edge	0.2262	0.6103	0.3301	4611
**Accuracy**			**0.8781**	93672

Supplementary Table 9 Metrics for the full pipeline - 65:35 split

**Full Pipeline (Stages 1 + 2) - Test Set**				
	**Precision**	**Recall**	**F1**	*Support*
Non-Crossing Edge	0.9828	0.8785	0.9261	77225
Crossing Edge	0.2193	0.6950	0.3334	3885
**Accuracy**			**0.8669**	81110
